# Designing drug shops for young women in Tanzania: applying human-centred design to facilitate access to HIV self-testing and contraception

**DOI:** 10.1093/heapol/czab084

**Published:** 2021-07-27

**Authors:** Lauren A Hunter, Sandra I McCoy, Aarthi Rao, Agatha Mnyippembe, Kassim Hassan, Prosper Njau, Rashid Mfaume, Jenny X Liu

**Affiliations:** School of Public Health, University of California, Berkeley, 2121 Berkeley Way #5302, Berkeley, CA 94720, USA; School of Public Health, University of California, Berkeley, 2121 Berkeley Way #5302, Berkeley, CA 94720, USA; Design and Innovation Lab, CVS Health, Boston, MA, USA; Health for a Prosperous Nation, PO Box 13560, Dar es Salaam, Tanzania; Health for a Prosperous Nation, PO Box 13560, Dar es Salaam, Tanzania; Health for a Prosperous Nation, PO Box 13560, Dar es Salaam, Tanzania; Ministry of Health, Community Development, Gender, Elderly and Children, Dodoma, Tanzania; Ministry of Health, Community Development, Gender, Elderly and Children, Dodoma, Tanzania; Institute for Health and Aging, Bixby Center for Global Reproductive Health, 490 Illinois Street, San Francisco, CA 94158, USA

**Keywords:** Human-centred design, adolescent health, HIV self-testing, contraception, drug shops

## Abstract

Adolescent and young adult women in sub-Saharan Africa experience barriers to sexual and reproductive health (SRH) services that elevate their risk of human immunodeficiency virus (HIV) acquisition and unintended pregnancy. Community drug shops may be effective distribution points to connect young women with SRH products. Thus, we used human-centred design (HCD) to create drug shops where young women could access HIV self-testing and contraception in Shinyanga, Tanzania. Enhancing the HCD process with behavioural science, we collected diverse data (i.e. 18 in-depth interviews, 9 ‘shadowing’ interviews, 6 shop observations, 6 focus groups) to understand the latent needs and motivations of young women and drug shopkeepers, brainstormed creative solutions and iteratively refined and tested solutions for acceptability, feasibility and cultural fit. We found a widespread moral imperative to control young women’s behaviour via misinformation about SRH, community gossip and financial control. Young women often engaged in mundane shopping at the behest of others. At drug shops, few SRH products were deemed appropriate for unmarried women, and many reactively sought SRH products only after engaging in higher risk behaviours. In response to these insights, we designed the ‘Malkia Klabu’ (‘Queen Club’) loyalty programme through which young women could earn mystery prizes by shopping at drug shops and discreetly request free SRH products, including HIV self-test kits, by pointing at symbols on loyalty cards. Our HCD approach increases the likelihood that the intervention will address the specific needs and preferences of both drug shopkeepers and young women. We will evaluate its effectiveness in a randomized trial.

**Key messages**

- Privately owned drug shops may be an effective distribution channel to engage adolescent and young adult women with sexual and reproductive health (SRH) services.
- We used human-centred design to generate novel strategies to reduce stigma and stimulate demand and create drug shops where young women can access HIV self-testing and contraception.
- Through this process, we designed the ‘Malkia Klabu’ (‘Queen Club’) loyalty programme through which young women can earn mystery prizes by shopping at drug shops and discreetly request free SRH products by pointing at symbols on loyalty cards.
- Our design approach increases the likelihood that the intervention will address the specific needs and preferences of both drug shopkeepers and young women.

## Introduction

Adolescent and young adult women (ages 15–24 years; hereafter ‘young women’) in sub-Saharan Africa face the dual threats of human immunodeficiency virus (HIV) infection and unintended pregnancy that severely undermine their long-term well-being. Young women comprise 25% of new adult infections in sub-Saharan Africa, are more than twice as likely to acquire HIV as their male peers in many countries and disproportionately bear 44% of all reported unintended births among women of reproductive age (Hubacher *et al.*, 2008; Fleischman and Peck, 2015; UNAIDS, The African Union, 2015; UNAIDS, 2016). Reasons for HIV and pregnancy prevention failures include stigma, lack of perceived risk and challenges accessing available health services (Michielsen *et al.*, 2010; Napierala Mavedzenge *et al.*, 2011; Nyblade *et al.*, 2017; WHO Regional Office for Africa, 2019). Social and cultural norms around young women’s behaviour may impede access. Young women seeking sexual and reproductive health (SRH) services may fear being perceived as promiscuous and face disapproval from parents, teachers, religious leaders and other community members (WHO Regional Office for Africa, 2019). In particular, biases against sexuality among young women by many health providers greatly discourage SRH care-seeking (Nalwadda *et al.*, 2011; Tumlinson *et al.*, 2015). Thus, despite the urgent need to reach young women with SRH services, public-sector health systems are ill equipped to overcome myriad access barriers alone.

### Moving beyond the health facility

Although HIV testing is freely available at public health facilities in Tanzania, nearly half of HIV-positive young women remain undiagnosed (TACAIDS, ZAC, 2018). Privately owned drug shops, including pharmacies, may be one effective distribution channel to engage young women with SRH services, including HIV self-testing and contraception. Located within communities and owned/staffed by community members, drug shops deliver over half of all healthcare in some low- and middle-income countries, at times providing consultations and diagnoses beyond their scope of practice at the behest of clients’ needs (Sudhinaraset *et al.*, 2013; Liu *et al.*, 2016). Pharmacies are important providers of SRH products, including emergency contraceptives and condoms, to adolescents and young adults (Gonsalves and Hindin, 2017; Gonsalves *et al*., 2020a,b). In an effort to increase access to quality-assured pharmaceuticals, Tanzania began the Accredited Drug Dispensing Outlet (ADDO) programme in 2003 to train and license retail shops to provide certain common medications, such as antimicrobials (TFDA, 2015; Embrey *et al.*, 2016). These drug shops are now located in nearly every community and vastly outnumber health facilities and pharmacies, offering unparalleled reach of health services to underserved populations in Tanzania (TFDA, 2015; Embrey *et al.*, 2016). Mostly small businesses frequently operated by women, ADDOs are often women’s first point of access for drugs, over-the-counter contraceptives, pregnancy tests and informal counselling and referral (Kagashe *et al.*, 2011; Rutta *et al.*, 2015; TFDA, 2015). Notably, although contraceptive services are free at public health facilities, 21% of women in Tanzania obtained their most recent contraceptive method from private sector drug shops (MoHCDGEC *et al*., 2016), and young women are more likely to rely on private sector method sources (Dennis *et al.*, 2017; SHOPS Plus, 2018).

With the recent introduction of HIV self-test kits into eastern and southern African markets, there is an imperative to leverage this opportunity and existing product distribution channels to increase testing coverage among vulnerable groups. OraQuick, the first HIV self-test kit to be prequalified by the World Health Organization, is an oral fluid screening test that displays high accuracy in the hands of lay users (WHO, 2016; 2020). Although HIV self-testing was legalized in Tanzania in 2019, HIV self-test kits are not yet registered or widely available outside of research settings (UNAIDS, 2019), but evidence from other countries in sub-Saharan Africa demonstrates that HIV self-testing is highly acceptable to youth, who value the increased convenience and confidentiality if affords (Indravudh *et al.*, 2017; Njau *et al.*, 2019; Pettifor *et al.*, 2020).

However, as evidenced by low uptake of contraception among young women in Tanzania (MoHCDGEC *et al*., 2016), merely making SRH products, including HIV self-test kits, available at drug shops is unlikely to engender wider adoption. Fears, misconceptions and stigma, among other cultural barriers, may obstruct uptake and reduce demand at these service delivery points. Thus, novel strategies to reduce stigma and stimulate demand are necessary to amplify the distribution of SRH services to young women in drug shops.

### Adopting a human-centred approach to programme design

Although myriad structural factors prevent young women from accessing SRH services, insufficient understanding of *individual* motivations, preferences and behaviours leaves many interventions unable to overcome the pressing daily concerns of young women that ultimately drive behaviours (Napierala Mavedzenge *et al.*, 2011). To fill this need and therefore develop acceptable and applicable interventions for demand creation, adopting a human-centred design (HCD) methodological process can yield strategies that are highly tailored and person centred (IDEO.org, 2015; McCoy *et al.*, 2017). HCD is a creative, empathetic approach for problem-solving that relies on rapid prototyping and testing of candidate solutions to develop informed and innovative solutions (IDEO.org, 2015). HCD evolved from private sector product innovation practices to develop creative products or services tailored to defined customer segments. In a departure from traditional qualitative approaches, HCD seeks to identify key insights to inform a specific intervention design rather than generalizable knowledge (Tolley, 2017). Accordingly, HCD draws on smaller numbers of participants than traditional qualitative methods but employs a wider range of data collection approaches and stakeholder groups to ensure both broad applicability and specific relevancy (Tolley, 2017). Furthermore, evidence-based strategies from behavioural economics (e.g. setting default options and using small incentives) can strengthen HCD-informed interventions by identifying opportunities to embed ‘nudges,’ or small alterations in how choices are presented, to further shape health decision-making (Dolan *et al.*, 2012). By deeply exploring the specific day-to-day interests, concerns and behaviours of relatively few individuals from diverse stakeholder groups, we hypothesize that HCD methods can capture individual and contextual richness that provides a strong foundation for behavioural interventions.

There has been increasing interest by the global health community in applying HCD to solve staunch public health challenges (Bazzano *et al.*, 2017; Holeman and Kane, 2020), including SRH challenges faced by young women. However, it remains underutilized in research contexts, in part due to perceptions of low transparency and reproducibility. Little is known about the systematic way HCD is being implemented, as few HCD-derived projects have made their way into the academic literature. In a scoping review of HCD’s applications in global health, Bazzano *et al*. (2017) noted ‘pervasive gaps […] related to replicable methods [and] description of methodologies used.’

Therefore, in this paper, we detail our application of HCD in an underserved population to shed light on this ‘black box’ and provide practical detail about an approach increasingly used in global health. We employed the established, iterative HCD process to develop a comprehensive intervention to motivate young women to obtain HIV self-testing and contraception at ADDOs, while simultaneously meeting the professional and financial needs of drug shopkeepers. By describing the process used to generate our intervention, we aim to highlight the potential for private industry design methods to tap into novel and unexpected strategies to improve health and well-being in low- and middle-income countries.

## Methods

### Design setting

Our study takes place in Shinyanga, a resource-limited, semi-rural region in Tanzania where we planned to newly introduce HIV self-testing in ADDOs. Most ADDOs in Shinyanga sell at least one contraceptive product (e.g. condoms, oral contraception, emergency contraception), with per unit prices typically ranging from 1000 to 6000 Tsh (0.43 to 2.59 USD).

In Shinyanga, HIV prevalence is 5.1% among young women, more than twice the prevalence among young men (2.0%), and 34% of women aged 15–19 years have begun childbearing (TACAIDS *et al*., 2013; MoHCDGEC *et al*., 2016), underscoring the importance of the proposed research.

Regular engagement with and support from national, regional and local stakeholders, including officials from the Ministry of Health, Community Development, Gender, Elderly and Children and the Pharmacy Council of Tanzania, were foundational to the design process.

### Design team

Our core design team comprised eight people: four Tanzania-based researchers who conducted all data collection activities described below (A.M., K.H., M.A., A.K.), three U.S.-based researchers (S.M., J.L., L.H.), and one U.S.-based design consultant (A.R.). The team was multidisciplinary, with expertise in behavioural science (J.L., S.M.), business administration (A.R.), epidemiology (S.M., L.H.), health economics (J.L.), human-centred design (A.R., A.M., K.H., S.M.), implementation research (S.M., J.L.), and marketing (A.M., K.H., M.A), as well as experience conducting health research at a local organization (A.M., K.H., M.A., A.K.).

Throughout the process, Tanzania-based and U.S.-based team members often convened separately to conduct in-person design activities, before converging remotely to share back, discuss, and identify next steps. However, all U.S.-based team members collaborated in-person with the Tanzania-based team members at least once during the design process, with in-person collaborations taking place across various phases: in preparation for beginning the design process (L.H., S.M.), during the first phase of data collection (J.L., A.R.), and during ideation and prototyping (S.M.). Before beginning data collection, each team member patronized a variety of drug shops in Shinyanga, often purchasing SRH products, to gain first-hand understanding of the shopping experience.

### Design process

**Figure 1.**
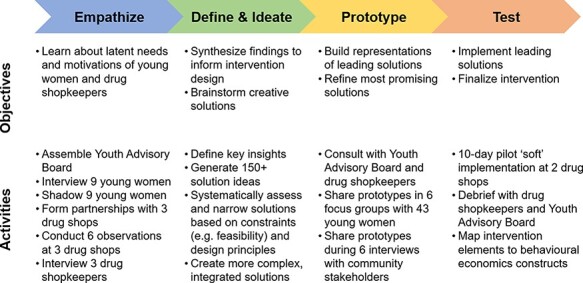
Objectives and activities during each phase of the human-centred design process

In the design process, we conducted a diverse set of formative research activities from September 2018 to June 2019 (Figure 1) to develop theoretically informed strategies to create drug shops at which young women—regardless of marital status—could access HIV self-testing and contraception. We employed an iterative process to generate, select, refine and integrate intervention components with many built-in check points throughout to ensure that the emergent intervention was on the right track and to generate preliminary signal to move forward into implementation. Note that in HCD, sample sizes are smaller and the data collection approaches and topics are intentionally much broader than traditional qualitative research as the goal is not theme saturation but rather to generate insights that provide a foundation from which to build context-specific solutions (Tolley, 2017). Detailed information about participant recruitment and data collection procedures is available in the Supplementary Appendix.

#### Empathize

We first sought to deeply understand the latent needs and motivations of young women and drug shopkeepers, how they interact with systems and how services can be better designed to build on existing behaviours and integrate seamlessly with their daily lives. We developed an information gathering strategy that included observing how young women and shopkeepers interact daily with their community.

To ensure that the research was centred around the views of young women, we assembled a Youth Advisory Board comprised of 19 local young women, between the age of 15–23 years, with whom we regularly consulted throughout the design process. The first meeting of the Youth Advisory Board focused on demonstrating the OraQuick HIV self-test kit, understanding young women’s baseline comfort and familiarity with SRH products available at drug shops (i.e. contraceptives and pregnancy tests) and gathering reactions and feedback on how to distribute HIV self-test kits and contraception at drug shops.

To understand the lived experiences of young women, female research team members conducted nine in-depth interviews and nine ‘shadowing’ interviews with women between the age of 16–24 years. Interviews were semi-structured and used a storytelling approach to understand young women’s experiences (e.g. asking ‘Can you tell me about the last time you visited a drug shop?’), and interviewers prioritized flexibility over reproducibility, focusing on topics on which participants had the most to say.

During ‘shadowing’ interviews, we followed participants for 8 hours during a typical day to observe social interactions, how their time and money was spent, their sources of trusted information and their primary daily concerns.

To elucidate shopkeepers’ perspectives, we partnered with three drug shops purposively selected to represent different shop sizes across different neighbourhoods (e.g. a small neighbourhood shop owned and operated by one person and a busy city-centre shop with several employees). We conducted six 3-hour structured observations (two per shop) to learn about daily shop operations and catalogue customer interactions with staff. We conducted in-depth interviews with three shopkeepers (two owners and one employee) using a semi-structured approach.

Interviewers took detailed notes during and after each interview and observation, which included emerging ‘pre-insight’ statements relaying key learnings about young women’s or shopkeepers’ experiences, needs and motivations. Interviews were also recorded and transcribed.

#### Define

We synthesized observations and interviews into insights. Whereas observations describe ‘what’ happened, insights are intended to capture the motivations underlying behaviour (i.e. answer ‘why’ questions) and illuminate opportunity areas for solutions. Each team member reviewed the interviewers’ written summaries of in-depth and ‘shadowing’ interviews and shop observations and independently generated observations and pre-insights, which were then shared through an in-person synthesis process: two teams of three (one Tanzania-based and one U.S.-based) met separately to share their observations and roughly group-related items (analogous to a ‘coding sort’ in qualitative research). Teams then collaboratively organized the notes into themes, which were gradually aggregated into initial insight statements. The two teams converged to discuss and finalize the insights and to identify emergent opportunities (i.e. unfulfilled needs that could be addressed by creative solutions).

The insights and opportunities formed the basis for solution generation. We generated three design questions as a platform for brainstorming solutions: (1) How might we encourage young women to go to drug shops? (2) How might we enable young women to obtain HIV self-test kits and contraception at drug shops? (3) How might we ensure that young women can use HIV self-test kits and contraception correctly? Simultaneously, we identified basic elements necessary to any solution model (e.g. shopkeeper training on the provision of HIV self-testing and contraception) before brainstorming novel strategies for addressing these design questions.

#### Ideate

We subsequently brainstormed creative solutions—both practical and ‘blue sky’—in multiple convenings of various sub-groups of the design team. To reduce group-think and encourage creativity, we employed a nominal group approach that begins with an individual round of brainstorming prior to group discussion to converge on the most compelling ideas (Delbecq and Van De Ven, 1971). During group discussions, ideas were vetted against practical constraints (e.g. limited physical space in drug shops), iteratively narrowed by the design team through voting based on feasibility and excitement and combined into more complex, integrated solutions.

#### Prototype

We iteratively prototyped leading solutions and tested them for acceptability, feasibility and cultural fit through several interconnected, concurrent data collection activities. We shared prototypes and gathered detailed feedback and suggestions during the second Youth Advisory Board meeting and one-on-one meetings with our three partnering shopkeepers. We held a series of six design-centred focus group discussions with young women to vet and refine prototypes of the proposed solutions.

Additionally, we conducted six in-depth interviews with purposively selected members of key community stakeholder groups (i.e. two parents of young women, two community officers, one pharmacist and one school head mistress) to better understand how the intervention could be received by the wider community. During these interviews, we solicited input on the feasibility and acceptability of leading solution ideas, potential implementation barriers, facilitating roles they could assume and strategies to create awareness about the availability of HIV self-test kits and other SRH products at drug shops.

#### Test

To confirm that the intervention was feasible in preparation for a larger evaluation study, we tested a simplified version of the programme through a 10-day ‘soft’ implementation at two of our partnering drug shops: one larger city-centre shop (which was often staffed by a male employee) and one small neighbourhood shop. This version of the programme did not include HIV self-testing, due to regulatory constraints, or emergency contraception, as this was not yet stocked by the small shop. Participating shopkeepers recorded young women’s engagement with the intervention, SRH product distribution and referral for facility-based contraceptive services. Research assistants visited the shops daily to review records and collect feedback from shopkeepers about the programme, including the reactions of their clientele and any potential adverse events. After the soft implementation, we finalized the intervention based on our debriefs with shopkeepers and the Youth Advisory Board.

To understand how the final integrated intervention addressed the needs of young women and shopkeepers, we took a step beyond the HCD process and categorized intervention elements according to the underlying behavioural economics construct that they aimed to leverage to identify the behavioural mechanisms (i.e. cognitive biases and ‘nudges’) underpinning the intervention strategy. This ensured that the intervention relied on multiple, evidence-based approaches for behaviour change that may increase its overall effectiveness.

### Protection of human subjects

All research activities were approved by the Tanzanian National Institute of Medical Research and the Human Research Protection Program at the University of California, San Francisco with the University of California, Berkeley Institutional Review Board in reliance. Participants provided written informed consent prior to interviews and focus groups; written parental consent and assent were obtained for participants under the age of 18 years.

## Results

Characteristics of participants in each phase are presented in Supplementary Appendix Table S1.

### Insights

The insights and corresponding opportunities are presented in Table 1, grouped according to their relevance to either young women (i.e. demand-side), shopkeepers (i.e. supply-side), or the intersection of the two (i.e. young women’s interactions with drug shops).

**Table 1.** Insights and opportunities defined during the human-centred process

	Insight	Observation (illustrative quote)	Opportunity
Adolescent and young adult women	**Surrounded by gatekeepers. There is a widespread moral imperative to control young women’s behavior.** During many interactions, people tell young women what to do or how to behave, reinforced by cultural norms around the community’s collective responsibility for child-rearing. People exert a gatekeeping role through misinformation and unsolicited advice, community gossip and cautionary tales, and financial control over young women’s spending.	‘*[My daughter] listens to my advice on whatever I tell her. I advise her on what she is supposed to do and what to ignore. Not to accept bribes because the guy might be an HIV victim. I also told her she might get pregnant and the guy might abandon her leaving her with a fatherless child and the child might end up in the streets.*’ —Mother of a young woman	Young women need freedom to make decisions based on their own needs and preferences.
	**I’m stuck. Young women are cash-strapped and have limited agency and mobility.** With little income or control over when they will get money or how they use their time, young women’s basic needs are often unmet. Young women must find small windows of opportunity or workarounds to move about and to get what they need.	‘*Sometimes I don’t have money at all, but I am in a relationship. If I tell him and he has some, he gives it to me.*’ — Young woman, age 22	Young women need access to convenient services that fit into the structures and constraints of their daily lives.
	**Mundane shopping. Shopping is typically a mundane chore that entails purpose-driven, fast interactions without browsing or comparison shopping.** As a result, young women often shop with pre-determined preferences and little consideration of new products.	‘*I am the one who sends [my daughter] to the shop, and I am the one who provides the money. So whenever I send her to the shop, I tell her to come back immediately.*’ —Mother of a young woman	Young women need to know about and be motivated to seek out a new product.
	**Contraception is a black box. Societal forces, including the educational system, impede young women’s knowledge of and access to contraception in order to exert control over young women’s sexual behavior.** Although young women feel that avoiding pregnancy is paramount to achieving their dreams, they fail to link their fear of pregnancy to concrete steps for prevention.	‘*In school, they taught us about contraceptive methods and their side effects. They told us it was best not to use them because there was a side effect for every benefit.*’ —Young woman, age 20	Young women need access to a reliable source of information about contraception.
	**Too little, too late. When it comes to their sexual health, young women seek out sexual and reproductive health (SRH) products only *after* engaging in risky behavior.** Constraints on their time, mobility, and financial resources impede proactive action. Young women may face more judgement and greater barriers to access when seeking preventative SRH products (e.g. condoms and other contraceptives) than when seeking SRH products related to the outcomes of sex (e.g. pregnancy and HIV tests).	‘*I was at home, and I missed my period. I told my aunt, and she told me to go buy a pregnancy test and instructed me on how to use it.*’ — Young woman, age 22	Young women need access to SRH information and products before engaging in sexual activity.
	**Too new to trust. HIV self-testing is exciting and confusing.** Many young women are eager to engage in HIV testing frequently and excited about HIV self-testing, but some question their ability to perform an HIV self-test correctly and have lingering concerns about the oral test’s validity.	‘*How can the test detect HIV in your saliva, if HIV is in your blood? Does this mean you can get HIV from kissing? Honestly, it is confusing.*’ —Young woman, age 18	Young women need to understand the test’s basic elements in order to trust the test.
Drug shopkeepers	**De facto doctors. Shopkeepers seek the recognition, confidence** **and power that comes with their association with healthcare professionals.** Some take pride in going above and beyond their drug dispensing duties to provide counselling and referral to customers. Despite this aspiration, their limited training may be revealed through poor advice, misinformation or errors in clinical judgement.	‘*We are like first aid before a person reaches the hospital. A person may be prescribed a certain medicine, but that medicine is not available at the hospital. When s/he brings me the hospital certificate, I will provide him or her with the required medicine. From this, I will have already given something to the community.*’— Shopkeeper	Shopkeepers need enhanced training and guidelines/tools to improve the quality of services and counselling that they provide to customers.
	**Competing goals. Shopkeepers feel that they are serving the greater social good, but this moral imperative is often conflated with their need to satisfy customer demand and make enough sales to be profitable.** They fulfil customers’ demand for what they want given what they can afford, even if it means sacrificing clinical quality in favour of profitability.	‘*Our businesses need professionalism and also convincing power. You saw the man who came: he wanted Malafin which costs 2,000 Tsh, but I convinced him to buy Duocotexin for 10,000 Tsh. Do you get me? If you have low convincing power, the business must shake.*’— Shopkeeper	Shopkeepers need to maintain clinical quality as a healthcare provider, but still satisfy customers’ demands
	**Business savvy to a point. Shopkeepers rely on a limited set of strategies to ensure their businesses’ survival, but largely fail to proactively innovate within their business.** Instead, they loosely follow the guidance of the government and distributors, even in the face of competition from larger pharmacies and other drug shops.	‘*In running this business, there isn’t anything very hard, if you follow the procedures given by Tanzanian Food and Drugs Authority. The issue here is to abide by their laws; that is it.*’— Shopkeeper	Shopkeepers need clear protocols on what to sell and training on business management, including advice about how to allocate their resources to maximize profit.
Intersection between young women and drug shops	**‘I go when I’m sick.’ Drug shops are largely quick in and out shops for acute over-the-counter needs triggered by unpleasant symptoms** Visits are intentional, and many customers go with a clear product, brand, and/or price in mind and are quick to go elsewhere should the shop fail to deliver. Some transactions occur with minimal communication and no negotiation.	‘*I just go to the drug shop to buy regular pills, if I have a headache or stomachache.*’— Young woman, age 22	(a) Young women need defined and compelling reasons to visit drug shops. (b) Shopkeepers need to satisfy customer demand in order to compete in a market over saturated with other shops.
	**Getting through the net. Shopkeepers exert paternalistic control in the name of altruism for young women, which may override their desire for sales.** Only some products are deemed acceptable for young women (e.g. pads, but not contraception), and consultative screening and questioning can be used to enforce shopkeepers’ gatekeeping role for SRH products as well as to determine clinical need.	‘*There are customers who ask for medicines that are not appropriate for them. Therefore, I start to ask about their ages and why they prefer to use this kind of family planning. I think injections are better for grown up women who have conceived several times.*’— Shopkeeper	Young women need privacy and safety from judgment to obtain sensitive products from drug shops and counselling that isn’t couched in moral judgement.

#### Adolescent girls and young women

We found a widespread moral imperative to control young women’s behaviour. Adults exert a gatekeeping role by continually instructing young women on what to do or how to behave. Young women are highly visible, attract unwanted attention and frequently run into people who interrogate and police their movements. Young women’s lack of privacy is further exacerbated by their cramped, shared living conditions at home and school dorms While family members are a primary source of social support and encouragement, they often also wield shame, cautionary tales and misinformation to influence young women’s behaviours. Young women must follow strict familial and/or school rules that dictate their access to money, ability to go places and make independent decisions. With few sources of income and limited access to money, young women must strategize to get resources from parents, boyfriends and friends, which can be unpredictable, sometimes leaving their basic needs (e.g. menstrual pads and soap) unmet.

For rural young women who rarely have personal spending money or decision-making power over their purchases, shopping is a mundane chore that entails little excitement or consideration of new products. Young women may visit shops and engage in small transactions many times per day, typically to purchase specific items from specific shops at the behest of others. When provided with money to buy something for themselves, some young women seem indecisive and stressed and may ask family or friends to help them decide what to buy. Beauty and hygiene products, like lotions and soaps that smell nice or a better brand of pads, are small luxuries that they value but often cannot afford. This makes risky solutions like transactional relationships with men more appealing.

In the face of societal barriers that limit young women’s access to reliable information, young women’s primary sources of contraceptive information are friends, partners and trusted adults. Information from these sources heavily influences their decision-making, but they have limited means to assess the quality of the information and must instead rely on the perceived trustworthiness of the messenger. Yet, teachers, relatives, and other trusted sources may use anecdotal stories, misinformation or withhold information to guide them away from contraception.

Consequently, preventing pregnancy feels outside of young women’s control, and they may fail to link their fear of pregnancy to concrete, proactive steps for prevention. Instead, many young women are reactive, seeking SRH products (e.g. emergency contraception) only after engaging in higher risk behaviour. In contrast to contraception, young women are bombarded with information about pregnancy and HIV, and pregnancy testing and HIV testing are normative behaviours that many young women engage in voluntarily (or are required to engage in at school). Some young women have more influence over whether partners test for HIV, emboldened by their own knowledge about HIV and increasingly conducive social norms around HIV testing. Many young women regularly test for HIV via blood-based HIV testing performed by healthcare professionals and are excited about oral HIV self-testing. However, there remain lingering doubts. Young women have varying levels of confidence in their ability to test alone, and since HIV is transmitted sexually and thought to live in the blood, some are sceptical about how it can be tested orally.

#### Drug shopkeepers

Drug shopkeepers are eager to be viewed as medical providers (although some may have little formal health training) and take pride in their role as the ‘first line’ of care in their communities. However, this role is often at odds with their profit imperative and their need to compete in a market saturated with drug shops. While shopkeepers seek to maintain some semblance of quality as a healthcare provider, they will satisfy customers’ demands, even if adherence to clinical standards suffers, in so far as it does not threaten their reputation. For example, some shopkeepers prefer to sell half-doses at the prevailing price rather than reduce prices, which may signal substandard product quality.

Despite competition from other shops, shopkeepers primarily follow the guidance of the government and distributors on which products to sell and do not proactively build upon or innovate within their business. Shopkeepers are often bored and have little to occupy their time while waiting for customers. Few shopkeepers make use of downtime between customers or innovate to increase customer traffic (e.g. opening on Sunday unlike other shops). In this way, they neglect opportunities to enhance their businesses.

#### Intersection between young women and drug shopkeepers

When young women visit drug shops, it is primarily for acute medication needs triggered by unpleasant symptoms. Most drugs shops do not tailor their offerings to attract students or young women and, thus, do not have interesting products for young women to explore. Products are placed behind the counter, necessitating proactive verbalization, which is often intimidating and can invite interrogation. Few SRH products are deemed acceptable for young women (e.g. pads, but not contraception), and unlike men, young women are often hassled when seeking sensitive products.

Although shopkeepers witness the SRH challenges facing young women in their communities first hand (e.g. through young women seeking HIV and pregnancy tests and abortifacients at their shops), they may not prioritize their social role in serving these needs. Rather, when faced with a choice about young women’s SRH, the balance is tipped toward social ideals and norms. Reinforced by their own beliefs about young women’s sexuality, shopkeepers can use misinformation to dissuade young women from getting what they want/need. Beyond determining clinical need, consultative screening and questioning can be used to enforce shopkeepers’ gatekeeping role for SRH products and to justify denying products to young women.

### Solutions

Informed by our insights, we identified basic elements necessary to any solution model, which centred in three categories: reliable information, SRH products and services, and privacy (Supplementary Appendix Table S2). We subsequently brainstormed creative solutions, collectively generating more than 150 solution ideas that were narrowed through voting (Supplementary Appendix Table S3 presents examples of discarded solutions). Concurrent with ideation, we developed design principles that defined aspirational goals for the solution, specifically how the solution must address the needs of both shopkeepers and young women across various domains (Supplementary Appendix Table S4). These principles were used to benchmark the potential of leading solutions for success. Eight solutions were subsequently prototyped, narrowed and refined in iterative focus groups with young women and through consultation with the Youth Advisory Board, shopkeepers and community stakeholders (Supplementary Appendix Table S5).

**Figure 2.**
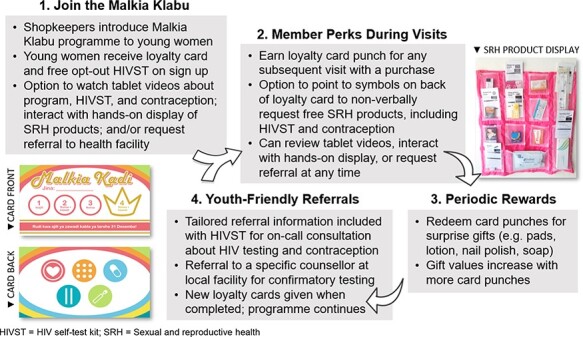
Components of integrated ‘Malkia Klabu’ intervention developed through the human-centred design process. HIVST = HIV self-test kit

The leading solutions were integrated into a loyalty programme called ‘Malkia Klabu’ (‘Queen Club’ in Swahili) (Figure 2), in which young women visiting drug shops can join to earn ‘mystery’ prizes. Upon joining, young women receive a loyalty card with colourful branding and a free HIV self-test kit as an opt-out sign-up gift, which includes tailored referral information (i.e. the first name and cell phone number of a trained, friendly HIV counsellor at a nearby health facility). Young women receive a punch on their card each day that they make a purchase at the drug shop and can redeem these punches to earn draws from one of two possible mystery bags which contain increasingly desirable items (e.g. soap and lip products in the first bag, and lotions and menstrual pads in the second bag).

On the back of the loyalty card are discreet symbols representing sensitive products available at the shop, namely HIV self-test kits, condoms, oral contraception, emergency contraception and pregnancy tests. When visiting the shop, club members can point to the product on the card that they want and receive it for free in a discreet bag without having to ask aloud.

To help shopkeepers deliver the programme, the rules are explained in a video available on a tablet, which also contains informational videos on using the HIV self-test kit and different contraceptive methods explained by young women (created by PSI Tanzania), which customers can watch using headphones for privacy. Each shop also has a hanging cloth display that contains sample SRH products (i.e. HIV self-test kit, pregnancy test and various short and long-term contraceptives), which customers can touch and explore, and simple informational cards about each contraceptive method’s effectiveness and potential side effects. Shopkeepers are trained to directly refer customers to a nearby youth-friendly health facility provider for longer acting contraceptive methods (i.e. injection, implant, IUD) included in the display.

Many of the programme elements correspond to evidence-based ‘nudge’ strategies from behavioural economics. For example, the loyalty programme aimed to introduce excitement (an *affect heuristic*) into young women’s shopping routine to further encourage visits to participating shops. Giving a free, opt-out HIV self-test kit upon club sign-up as a *default* feature helps ensure uptake, and being able to earn small-value mystery prize incentives is intended to overcome tendencies to undervalue the importance of SRH prevention (*present bias*), encouraging uptake at the moment of interaction. For shopkeepers, the programme seeks to underscore their desired status as a community health champion (*ego*) stemming from their eagerness to be viewed as medical providers who take pride in their role as the ‘first line’ of care in their communities. Further, given that shopkeepers were observed to primarily follow the guidance of the government and distributors on which products to sell, being a member of an exclusive group of specially branded shops (*peer effects*) signing on to deliver a programme officially sanctioned by local authorities reinforces their *commitment* to health provision for a vulnerable population.

### Testing

During the soft implementation, 41 young women who visited the two shops over 10 days were offered club membership by the shopkeepers. Of these, 39 signed up, and 23 completed a return visit within 16 days of the start date to earn a free prize from a mystery box. In total, club members requested and were provided with 13 packs of condoms, 16 pregnancy tests and 7 packs of oral contraception for free. Shopkeepers provided 11 referrals to facility-based contraceptive services (e.g. the injection, implant or IUD). Although we did not collect data on referral success, one shopkeeper reported that a young woman later returned to the shop and confirmed that she received the injection at the health facility to which she was referred. No adverse events were reported. However, one shopkeeper received negative feedback from an adult male customer, who objected to teaching young women about contraception on religious grounds due to concern that it would encourage sexual activity.

Overall, both participating shop owners reported high satisfaction with the programme, stating that it increased traffic and that customers of all ages enjoyed engaging with the contraceptive display and videos. The Youth Advisory Board also expressed excitement for the programme and provided feedback to refine implementation, such as redesigning the card to make the symbols on the back stand out more clearly and adding condom brands of different prices in the SRH product display.

## Discussion

Young women in Tanzania regularly patronize community drug shops where SRH products are available, but pernicious barriers prevent many from obtaining these products. We hypothesized that addressing these barriers requires innovative strategies to increase demand that diverge from business-as-usual approaches—a challenge that required a similarly innovative methodological process. By adopting elements of private industry product innovation to our specific objective of increasing access to SRH products for young women in drug shops, we learned four valuable lessons:

*(a)* *An immersive, empathetic approach starting from a deep understanding of contextual factors influencing young women’s interactions with drug shops led to unique insights critical to intervention development that may have otherwise been missed in traditional qualitative research.*

By taking a holistic view of target users’ needs and preferences, the HCD approach may help to avoid narrow intervention strategies that are largely dissociated from the non-health-related motivations and influences of people’s daily lives (Mavedzenge *et al.*, 2014; Chandra-Mouli *et al.*, 2015). For example, we learned that shopping is often a mundane chore that does not allow for product exploration and that young women are eager to touch and feel products. As such, the loyalty programme capitalizes on young women’s frequent visits to shops while prioritizing discretion to enable young women to engage with and obtain SRH products at participating drug shops. Crown imagery and colourful design of the loyalty card, product displays and small-value mystery prize incentives introduce excitement into routine shopping. Hence, while ‘shopping’ does not seemingly fit with what researchers usually focus on, care-seeking for health has many parallels, and the user experience should be addressed from a more holistic service perspective—from home or school to the market and shop—rather than just from a clinical point of view.

While HCD, by definition, tends to follow a similar process of empathizing with the target user and cycles of rapid prototyping and testing, the specific activities may vary; the process described here provides only one example that can be modified or built upon as needed. Unlike traditional research methods, the goal is not replicability of results: there are many creative strategies that may address a given objective in a specific setting, and thus, each design process may produce different results.

*(b)* *By grounding the design process in the experiences of both young women and shopkeepers, we created a programme that fit within the constraints of young women’s day-to-day lives and shopkeepers’ business practices and that was highly acceptable to both.*

Our design challenge entailed creating an intervention for the intersection of demand *and* supply or where young women meet shopkeepers. Although interventions creating ‘youth-friendly’ environments show potential for promoting service uptake, no interventions have yet harnessed the crosscutting role that drug shops play in supplying products (e.g. cosmetics and menstrual hygiene) and health commodities (e.g. contraception and pregnancy tests) that young women demand, while also serving as a gateway to clinical services, including for HIV. Our study bridges these gaps by engaging drug shops to specialize in delivering SRH services, including HIV self-testing and contraception, to young women.

To that end, we sought to understand the latent needs and preferences of two key stakeholder groups: young women and shopkeepers. In our solution development, several intervention features were included to align the interests of shopkeepers with young women’s needs. Malkia Klabu incentivizes shopkeepers to cater to young women by requiring purchases from members (whether participants come to the shop to purchase things with their own money or while running errands for others) to earn loyalty card punches, increasing shops’ financial security and building a loyal customer base of young women. The added draw of mystery prizes involving beauty and hygiene products increases the array of products young women are interested in. Using the symbol card enables young women to ask for SRH products without embarrassment and interrogation, while also giving shopkeepers implicit permission to offer these products to young women rather than restricting their access. Product displays with freely accessible information facilitate semi-private independent exploration to correct misinformation that young women may receive from trusted friends, adults and partners. Our approach, thus, increases the likelihood that the final intervention will be relevant, salient and acceptable to young women and shopkeepers and ultimately sustainable.

*(c) The HCD process was enhanced by leveraging* *behavioural* *economics ‘nudges’ (e.g. incentives, commitments, defaults* *and* *saliency) in the final intervention to ensure that design features incorporated evidence-based motivational elements.*

This intervention builds upon young women’s and shopkeepers’ existing behaviours and leverages a variety of evidence-based ‘nudge’ strategies from behavioural economics to support decision-making over SRH product uptake. Research has demonstrated that such nudges can effectively overcome barriers to uptake across various health behaviours and contexts for both one-time actions (e.g. HIV testing and clinic visits) and those requiring longer fidelity (e.g. drug adherence and prevention of mother-to-child transmission) (McCoy *et al.*, 2017; Sibanda *et al.*, 2017).

Notably, our approach incorporates multiple nudge strategies simultaneously, which precludes our ability to assess the independent effects of different, singular strategies associated with one intervention design element. However, we adopted this approach to ensure that the intervention could resonate with a variety of young women and shopkeepers and reduce as much friction as possible in the experience of obtaining and providing SRH products. We recognize that young women themselves and their surrounding environments are highly fluid during these critical years of development as they transition toward independent adulthood. Thus, there may not be any one single ‘magic bullet’ strategy. Rather, multiple complementary strategies motivating SRH prevention increase the likelihood that the resulting intervention is salient to a wide variety of young women. While the intervention was carefully designed and tailored to the local context, intentionally embedding behavioural strategies also enables the programme to be readily adapted to a different context; the same motivational elements can be retained while their execution (e.g. specific imagery or videos) may be adjusted.

*(d)* *Interventions designed within environments with restrictive social norms may motivate change at the individual level through reinforcements built into everyday interactions.*

Importantly, our design challenge involved developing an acceptable, feasible, appropriate and potentially scalable solution for SRH prevention among young women—a global health priority for which little progress has been made despite decades of efforts. Restrictive social norms about young women’s sexuality at a time when they are increasingly learning to become independent permeate all layers of the environment in which young women live, even from their most trusted friends, partners and family members who may relay misinformation or cautionary tales which discourage contraceptive use.

These insights highlighted an opportunity for leveraging interest and knowledge around HIV testing and more acceptable social norms surrounding HIV and pregnancy testing to influence norms around contraception. Several programme features work in concert to normalize care-seeking and uptake of contraceptive products. Malkia Klabu engages young women at their first interaction with a free opt-out HIV self-test kit upon sign-up and then allows them access to free contraceptive products at any visit. Enabling non-verbal communication with the symbol card signals explicit and implicit permission to ask for contraceptives if desired without fear of interrogation. The applicability and value of the information conveyed is heightened by creating a sense of belonging vis-à-vis exclusivity of the programme to young women and providing informational videos featuring similar young women using the programme or contraceptive products.

### Future directions

By following the HCD process, we developed a tailored intervention that was feasible to implement in drug shops and highly acceptable to young women and drug shopkeepers. During the next phase of this study, we will apply traditional epidemiologic research methods to formally evaluate the Malkia Klabu intervention. Specifically, we will assess the intervention’s preliminary effects through a 4 month randomized trial at 20 drug shops. We will measure patronage among young women, distribution of HIV self-test kits and contraception and health facility referrals and evaluate intervention effectiveness, acceptability, potential risks and adverse events and implementation strengths and weaknesses using a rigorous, mixed-methods approach. The trial has been preregistered at ClinicalTrials.gov (NCT04045912). This trial will determine whether a larger study is warranted to assess downstream impacts (e.g. HIV diagnosis and unintended pregnancy), explore strategies for scalability and sustainability (e.g. pricing, supply chain) and evaluate whether the intervention translates to larger urban settings in Tanzania.

## Supplementary Material

czab084_Supp

## Data Availability

The data underlying this article cannot be shared publicly to protect the privacy of participating individuals but may be shared on reasonable request to the corresponding author.
