# Supplementary material for: Designing drug shops for young women in Tanzania: applying human-centred design to facilitate access to HIV self-testing and contraception
Source: Health Policy Plan. 2021 Jul 27;36(10):1562–73. doi: 10.1093/heapol/czab084 (PMC8597958; doi:10.1093/heapol/czab084)
Supplement: czab084_Supp [file czab084_supp.zip › Supplementary Appendix - HPP.docx]

**Supplementary Appendix**

Designing drug shops for young women in Tanzania: Applying human-centred design to facilitate access to HIV self-testing and contraception

[Recruitment and data collection procedures 2](#_Toc77816555)

[Table S1. 4](#_Toc77816556)

[Table S2. 5](#_Toc77816557)

[Table S3. 5](#_Toc77816558)

[Table S4. 6](#_Toc77816559)

[Table S5. 7](#_Toc77816560)

# Recruitment and data collection procedures

- **Youth Advisory Board:** Potential board members were identified by local community development officers and were asked to bring a friend to the first meeting (snowball recruitment).
- **In-depth interviews and ‘shadowing’ interviews with adolescent and young adult women:** We identified participants through referrals from local research staff, community development officers, and other study participants, and purposively sampled them to achieve diversity in age and educational status. In-depth interviews covered a broad variety of topics (e.g. relationships with family, friends, and romantic partners; experiences at drug shops and healthcare facilities; pressing sexual and reproductive health [SRH] concerns; perceptions of SRH products; and reactions to a hands-on HIV self-test kit demonstration).

Seven ‘shadowing’ interviews (hereafter, ‘shadows’) were conducted in person, while another two used a video-based approach intended to increase privacy and encourage candor. During video-based shadows, participants were given a camera with which to record video clips of themselves describing their experiences, interactions, and emotions as they went about their day. During six shadows (5 in-person, 1 video-based), we additionally provided participants with a small amount of their participant compensation (~USD2) for them to spend as desired to directly observe their real-world shopping behaviours and preferences.

To mitigate the extent to which participants altered their responses and/or behaviour while being observed (i.e. Hawthorne effect), data collection activities with young women were conducted by trained interviewers who were themselves Tanzanian women in their 20s. Additionally, data collection efforts often focused on observing and understanding mundane aspects of young women’s day-to-day behaviour, some of which may be less prone to misreport or alteration.

- **In-depth interviews with shopkeepers:** All three drug shops coincidentally had female shopkeepers, in part reflecting the preponderance of female-run Accredited Drug Dispensing Outlets (ADDOs) (TFDA, 2015). Interviews focused on shopkeepers’ perceptions of young women and their health needs; roles in community-based health provision; views on distributing HIV self-test kits, contraceptives, and other young woman-centred merchandise (e.g. skincare and menstrual hygiene products); and reactions to an HIV self-test kit demonstration.
- **Focus groups with adolescent and young adult women:** Focus group participants were referred by Youth Advisory Board members and previous participants and were purposively selected to represent young women in and out of school. In each focus group, participants engaged in fun, interactive role plays and/or simulated drug shop interactions with physical mock-ups of solution elements. Participants provided detailed feedback and suggestions in group discussions and anonymously voted on and/or rated solution elements. This information was used to determine which solutions were potentially motivating, feasible (given shop operational constraints), and could be seamlessly incorporated into young women’s daily routines. Activities in later focus groups became increasingly specific as intervention elements were narrowed and refined, with participants identifying preferred features and/or specific design elements (e.g. branding, symbols, colours).

Table S1. Participant characteristics by phase of the human-centred design process.

|  | **Participant category** | **Characteristics** | **N** |
| --- | --- | --- | --- |
| **Phase 1: Empathize (n=18)** | Adolescent and young adult women | Age  15-19  20-24 | 6  9 |
|  |  | Educational status  Primary school only  Enrolled in secondary school | 7  8 |
|  |  | Activity*  In-depth interview  In-person ‘shadowing’ interview  Video-based ‘shadowing’ interview | 9  7  2 |
|  | Drug shopkeepers** | Employee type  Owner  Dispenser | 2  1 |
|  |  | Age  30-39  40-49  60-69 | 1  1  1 |
| **Phase 3: Prototype (n=49)** | Adolescent and young adult women | Age  15-19  20-24 | 35  8 |
|  |  | Educational status  Primary school only  Enrolled in secondary school | 25  18 |
|  |  | Activity  Focus group 1  Focus group 2  Focus group 3  Focus group 4  Focus group 5  Focus group 6 | 7  4  6  8  10  8 |
|  | Community stakeholders*** | Sex  Male  Female | 3  3 |
|  |  | Age  30-39  40-49  50-59 | 2  1  3 |
|  |  | Role  Community officer  Health facility pharmacist  Mother of young woman  Secondary school headmistress | 2  1  2  1 |

*Two participants completed both an in-depth interview and a ‘shadowing’ interview. One participant completed two ‘shadowing’ interviews (1 in-person, 1 video-based).

**All participating drug shopkeepers were female. All participating shopkeepers completed an in-depth interview, and researchers conducted two 3-hour observations at their drug shops (6 observations in total).

***All participating community stakeholders completed in-depth interviews.

Table S2. Necessary elements of any solution model defined in Phase 2 (‘Define & Ideate’) of the human-centred design process.

| **Category** | **Necessary solution elements** |
| --- | --- |
| Reliable information | - Training for shopkeepers on providing HIV self-testing and contraceptive services to young women - Hands-on product displays at drug shops with information on HIV self-testing and contraception |
| SRH products/services | - Free provision of HIV self-test kits and contraceptives to young women at drug shops - Tailored referral information included with HIV self-test kits for on-call consultation about HIV testing and/or contraception |
| Privacy | - Discreet packaging for HIV self-test kits and other SRH products - Option to anonymously dispose of used HIV self-test kits at drug shops |

SRH = Sexual and reproductive health

Table S3. Example solution ideas discarded during Phase 2 (‘Define & Ideate’) of the human-centred design process.

| **Solution idea** | **Reason for discarding** |
| --- | --- |
| Have private space for young women at drug shops (e.g. storage lockers or spaces to socialize) | Low feasibility due to space constraints |
| Host special community events for young women at drug shops (e.g. beauty events, movies, educational and vocational classes, back-to-school events) | Low feasibility due to space constraints and young women’s constrained mobility |
| Connect drug shops with schools or churches as a preferred provider of school supplies and other products | Low excitement |
| Create a layaway programme where young women can get products they need (e.g. pads) after completing payments | High financial risk for young women and shopkeepers |
| Have a bin from which young women can grab SRH products without interacting with shopkeepers | Low feasibility due to shopkeepers’ preference for having control over products |
| Train peer ambassadors to educate other young women (e.g. at home events or via SMS/phone calls) | Information-only programme unlikely to lift other key constraints to SRH products |
| Relay information about SRH products via a young woman-friendly cartoon, magazine, or newsletter provided at drug shops | Low sustainability due to resource demands to maintain fresh content |
| Provide shopkeepers with badges and rewards based on quality of services (e.g. using mystery audits or customer feedback forms) and/or completion of special training activities/quizzes | Low excitement |

SRH = Sexual and reproductive health

Table S4. Design principles defined in Phase 2 (‘Define & Ideate’) of the human-centred design process.

|  | **The solution must…** | |
| --- | --- | --- |
| **Domain** | **Adolescent and young adult women** | **Shopkeepers** |
| Larger aspirations | - Have a tangible connection to aspirations of self-sufficiency, independence, financial security, and positive reputation | - Affirm shopkeepers’ role/reputation in the community as a health worker - Improve financial security |
| Emotional state | - Trigger curiosity - Build confidence and SRH knowledge | - Ensure that shopkeepers are informed, capable, and confident |
| Physical space/access | - Create an environment that is welcoming, hands-on, private, and judgement-free - Ensure that services are convenient and accessible within young women’s constraints (mobility, time, financial) | - Allow shopkeepers to secure and maintain control over their products - Fit in a small space |
| Products | - Enable young women to access contraception and HIV self-testing a low cost - Include products that are exciting (e.g. beauty products, desirable pad brands) | - Promote sufficient turnover (sales) - Attract customers - Have acceptable procurement costs |

SRH = Sexual and reproductive health

Table S5. Leading solutions prototyped in Phase 3 (‘Prototype’) of the human-centred design process.

| **Name** | **Description** | **Status** |
| --- | --- | --- |
| Basic bundle | With any purchase, young women are given a free HIV self-test kit (opt out). | Dropped due to low excitement |
| Peer bundle | With any purchase, young women are given two HIV self-test kits, one to give to a friend. | Dropped due to reluctance to offer HIV tests to friends |
| Peer referral | Young women can earn a free gift if they have three friends come into the shop. Young women who sign up are given a card with their name on it to redeem their gift. The shopkeeper writes down the woman’s name in order to check off how many times others come in and give her name. | Dropped due to low acceptability of name-based tracking programmes |
| Subscription service | Young women join a club to receive free pads for the next two months. To join, they must purchase the first pack of pads up front, paying slightly more than the original cost. Members receive a free HIV self-test kit as a sign-up perk and are given a membership card with the dates on which they can return to the shop for next two packs of pads. Members return each of the following two months to pick up a new package of free pads. | Dropped due to low feasibility with young women’s financial constraints |
| Delivery service | Young women are given a card that has a phone number that they can call and/or SMS to request any product that they want for delivery. SRH products have codenames, which are also listed on the card. Products are packaged privately so that the delivery person does not see the contents.  Option A: Young women call the shopkeeper, who has products delivered by a professional delivery company.  Option B: Young women call the shopkeeper and/or selected motorcycle taxi drivers to have products delivered via motorcycle taxi.  Option C: Young women from the community are given a bicycle and paid to act as delivery persons. Other young women contact them directly to ask them to pick up products for them. | Dropped due to low excitement and logistical constraints (e.g. requires access to phone) |
| Basic loyalty | Young women sign up for a loyalty programme and receive a free HIV self-test kit as a sign-up gift. Members receive a stamp for each visit to a drug shop in which they make a purchase. Once enough stamps are earned, members earn a free pack of pads. | Dropped due to low excitement |
| Loyalty plus | Young women sign up for a loyalty programme and receive a free HIV self-test kit as a sign-up gift. Members receive a stamp for each visit to a drug shop in which they make a purchase. Once enough stamps are earned, young women draw from increasing valuable mystery boxes containing desirable products (e.g. soap, lotion, pads).  Option A: Members buy individually packaged pads with small amounts of pocket change and receive a stamp for each pad that they buy. After eight pads are purchased, they earn a draw from the mystery box.    Option B: Each time members buy a package of pads, they earn a stamp and get to draw from the mystery box.  Option C: Each time members make any purchase, they receive a stamp and may earn a draw from the mystery box. | Option A dropped due to low acceptability among young women due to hygiene concerns  Option B dropped due to low feasibility with young women’s financial constraints  Option C integrated in final intervention |
| Symbol card | Young women are given a card with discreet symbols that represent HIV self-test kits, pregnancy tests, and contraceptives available in drug shops. When shopping, young women point to the products that they want on the card to have them given to them for free without having to ask aloud. | Integrated into final intervention |

SRH = Sexual and reproductive health
